# Locomotion Training in a Care Prevention Program for Community-Dwelling Older Women in Japan: An Exploratory Pre-post Intervention Study

**DOI:** 10.7759/cureus.101393

**Published:** 2026-01-12

**Authors:** Ikki Yoshida, Yohei Sawaya, Tamaki Hirose, Yuta Hanawa, Masahiro Hirose, Kiyoshi Ueda, Hiroto Ito, Tomohiko Urano

**Affiliations:** 1 Department of Rehabilitation, International University of Health and Welfare Shioya Hospital, Otawara, JPN; 2 Department of Physical Therapy, School of Health Sciences, International University of Health and Welfare, Otawara, JPN; 3 Department of Rehabilitation, International University of Health and Welfare Shioya Hospital, Yaita, JPN; 4 Department of Rehabilitation, International University of Health and Welfare Hospital, Nasushiobara, JPN; 5 Department of Geriatric Medicine, School of Medicine, International University of Health and Welfare, Narita, JPN

**Keywords:** japan, locomotion training, locomotive syndrome, older adults, women

## Abstract

Introduction

This study assessed changes in locomotive syndrome (LS) risk test scores following locomotion training (LT) in community-dwelling older Japanese women participating in a local government care prevention program and provided evidence for its effectiveness and dissemination.

Materials and methods

This interventional study employed a pre-post design and was conducted between September and December 2024. The participants were 14 women (mean age: 76.5 ± 4.5 years) who participated in a long-term care prevention program. A 76-day exercise intervention consisting of LT was implemented. LS was assessed pre- and post-intervention using the stand-up test, the two-step test, and the 25-item Geriatric Locomotive Function Scale (GLFS-25).

Results

Three participants who were not classified as having LS pre-intervention remained in the non-LS category. Among the 11 participants who had LS pre-intervention, four individuals (36.4%) showed improvement in their LS stage, while seven (63.6%) remained in their LS stage. No participant experienced worsening of their LS stage. The GLFS-25 score improved significantly post-intervention [pre-intervention: median 6.5 (interquartile range: 3.0-14.3); post-intervention: median 4.5 (2.0-9.3); p = 0.011].

Conclusions

As a program conducted within the limited time and resources of preventive care projects, LT may be suggested as an effective exercise intervention for improving musculoskeletal function in older individuals.

## Introduction

There is a rapid increase in the aging population in Japan. In 2023, the number of adults aged 65 years and older exceeded 30 million, representing nearly one-third of the total population [1]. With advancing age, the number of people certified as requiring support or long-term care has been steadily increasing every year, and the rising medical and nursing care costs have become serious social issues [2]. The main reasons for the need for support or long-term care include diseases related to the musculoskeletal system, such as joint disorders, fractures, and falls [3]. With the ongoing aging of society, it is necessary to prevent musculoskeletal functional decline to maintain the independence and quality of life (QOL) of older individuals.

Locomotive syndrome (LS), proposed in 2007, is a recently focused concept defined as “a condition in which the mobility function declines due to impairments in the musculoskeletal system” [4]. The assessment of LS was progressively refined with the introduction of the risk test in 2013, the establishment of clinical thresholds for LS stages 1 and 2 in 2015, and the addition of LS stage 3 in 2020. LS has been reported to be associated with the health-related QOL [5,6], decreased activities of daily living (ADL), increased risk of falls [7], and cognitive decline [8]. Additionally, LS is related to various musculoskeletal disorders, such as osteoporosis, osteoarthritis, and spinal diseases, and approximately 47 million Japanese individuals aged ≥40 years are estimated to be at risk for LS [9]. LS is a major factor contributing to new certifications for long-term care in older adults, accounting for approximately one-quarter of such cases [9,10], and its prevention and early intervention are important issues that contribute to extending healthy life expectancies and controlling medical and nursing care costs.

Locomotion training (LT) is an exercise program proposed by the Japanese Orthopedic Association that aims to prevent and improve the progression of LS [3,4]. LT is a simple exercise that can be easily incorporated into daily life without an undue burden. It consists of two basic exercises: “single-leg standing” for balance training and “squats” for lower-limb muscle strengthening. LT can be performed while holding onto a support, such as a handrail, as it is safe and accessible even for older adults with reduced physical function, making it easier to continue regularly. Although LT is widely recommended, studies on its effectiveness according to the frequency of implementation and characteristics of the target population remain insufficient. Accumulating scientific evidence regarding the effects of LT interventions is an urgent issue in the field of orthopedics in Japan.

This exploratory study aimed to examine post-intervention changes in musculoskeletal function resulting from LT among community-dwelling older women participating in a preventive care program implemented by a local government. It also sought to identify the characteristics of older individuals who may benefit more from LT and to provide preliminary evidence supporting the usefulness and dissemination of future interventions for LS prevention.

## Materials and methods

Study design and ethical considerations

This interventional study used a pre-post design, with an intervention period spanning from September 27, 2024, to December 13, 2024. This study was approved by the Ethics Review Board for Clinical Research of the International University of Health and Welfare (approval number: 24-TC-008) and registered in the University Hospital Medical Information Network (UMIN) clinical trial registry (Trial ID: UMIN000055631). Ethical conduct was ensured in accordance with the Declaration of Helsinki. Before taking part in the study, participants were fully informed about the purpose and procedures of the research and gave their written consent.

Participants

**Figure 1:**
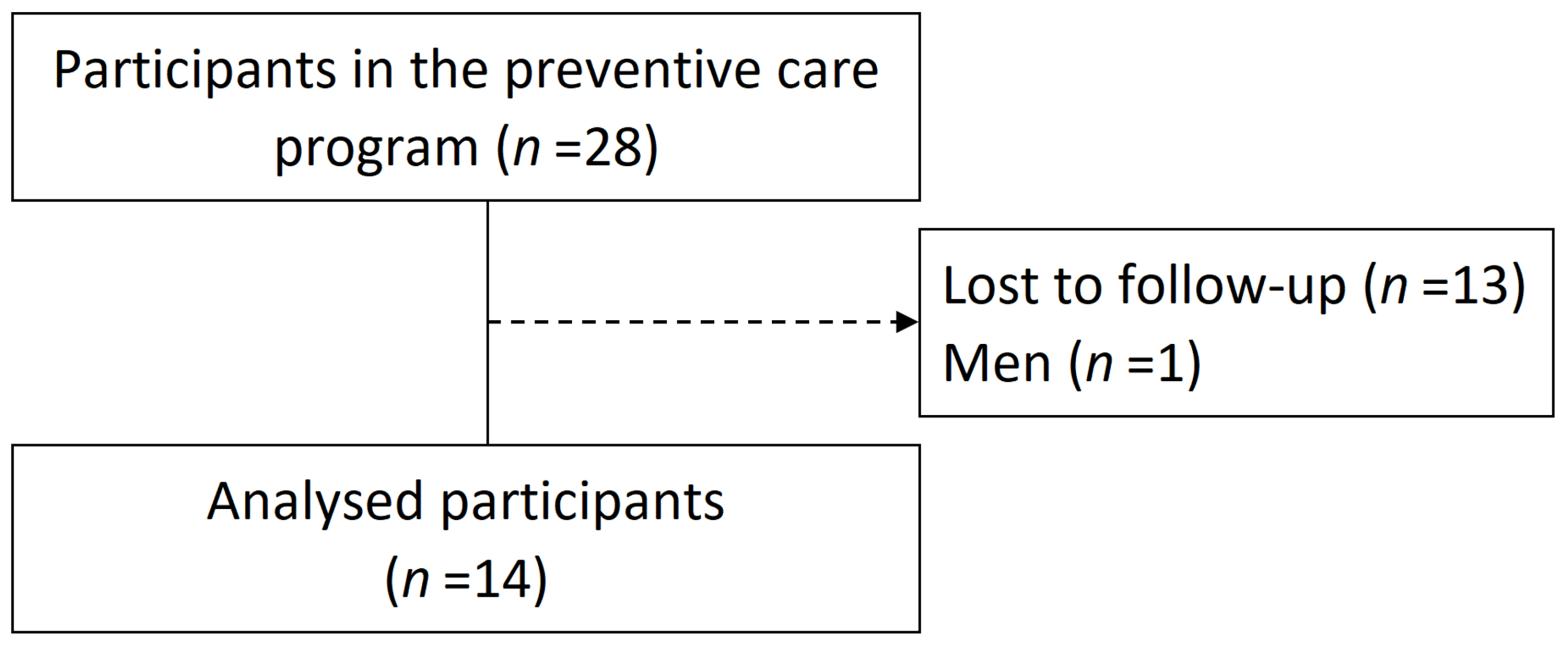
Flow chart of the study participants

The participants were 28 individuals who attended a preventive care class in Yaita City, Japan, between September and December 2024. Of these, 13 participants who did not attend the post-intervention measurement session and one male participant were excluded, resulting in a final analysis set of 14 female participants (mean age: 76.5 ± 4.5 years; Figure 1). None of the participants had comorbidities that would interfere with the assessments, and all participants were able to safely perform LT exercises without complications. In addition, none of the participants used walking aids such as canes or walkers.

The participants in the preventive care class were recruited through the official website of Yaita City, Japan. Prior to the intervention, evaluations of LS and frailty were conducted for all participants, and a physical therapist provided standardized instructions for LT. Similar evaluations of LS and frailty were conducted following a 76-day LT intervention to assess pre- and post-intervention changes.

Locomotion training

The LT proposed by the Japanese Orthopedic Association consists of two exercises: single-leg standing and squats.

Single-Leg Standing

Single-leg standing has been reported to improve the balance and coordination of the trunk and lower limb muscles, contributing to a reduced risk of falls [11]. The implementation method involved raising one foot 5-10 cm off the ground with the eyes open. Exercise was performed for one minute on each side, three times per day [3]. Additionally, to prevent falls, participants were instructed to perform the exercise while holding onto a wall, table, handrail, or chair [12].

Squats

Squats are bodyweight training exercises that strengthen the major lower limb muscle groups, such as the quadriceps femoris, gluteus maximus, and hamstrings [13]. In addition to improving balance ability, squats have been reported to be effective for improving ADL [14]. The exercise method involved standing with feet shoulder-width apart, bending the knees slowly while keeping the knees from moving past the toes, lowering the hips, and returning to a standing position repeatedly [3]. The participants were also instructed not to bend their knees beyond 90° while lowering their hips. The prescribed exercise volume was five to six repetitions per set, performed in three sets per day.

LT implementation status

To understand the continuation status of LT, calendar-style record sheets were distributed at the time of LT instruction, before the intervention. The participants were instructed to record their daily exercise performance according to the following criteria: days when LT was performed as prescribed (squats: three sets of five to six repetitions; single-leg standing: one minute on each side, repeated three times) were marked with “circle, performed as instructed”; days when the exercise was partially performed with at least one repetition, but lesser than the prescribed amount were marked with “triangle, partially performed”; and days when the exercise was not performed at all were marked with “X mark, not performed.” The participants recorded the corresponding symbols in the data column. In addition, staff members verbally confirmed the implementation status once a month. The LT intervention period lasted 76 days, during which the participants performed the LT exercises at home.

Assessment

LS was assessed before and after the intervention using the LS risk test, which consists of three components: the stand-up test, the two-step test, and the 25-item Geriatric Locomotive Function Scale (GLFS-25) [3,15].

In the stand-up test, the participants attempted to rise from stools 20, 30, and 40 cm in height using either one or both legs. If a participant was able to stand up from a 40-cm stool using both the left and right legs individually, they were classified as not having LS. If they failed to stand up from a 40-cm stool on either the left or right leg, but succeeded in using both legs from a 20-cm stool, they were classified as having LS stage 1. Participants who could not stand from the 20-cm stool using both legs, but could stand from the 30-cm stool, were classified as having LS stage 2. If they were unable to stand from the 30-cm stool using both legs, they were classified as LS stage 3 [15-17].

In the two-step test, the participants aligned their toes with the start line, took two maximal steps forward without losing balance, and brought their feet together. The two-step test was expressed as the ratio of the total step length (cm) to the participant’s height (cm). The value of 1.3 or higher was classified as not having LS; a value of ≥1.1 and <1.3 was classified as LS stage 1; a value of ≥0.9 and <1.1 as LS stage 2; and a value of <0.9 as LS stage 3 [15,17].

The GLFS-25 is a self-reported scale with 25 items used to assess physical, psychological, and social problems. Total scores spanned a range of 0-100, with increasing values reflecting greater impairment in mobility and daily functioning. A score of <7 was classified as not having LS; a score between 7 and 15 as LS stage 1; between 16 and 23 as LS stage 2; and 24 or higher as LS stage 3 [15,17,18].

The overall LS classification was assigned according to the poorest performance observed across the three assessments.

Frailty

Frailty was assessed before and after the intervention by using the Questionnaire for Medical Check-up of the Old-Old (QMCOO). The questionnaire consisted of 15 items covering 10 domains: general health status, mental health, dietary behavior, oral function, weight loss, physical function, falls, cognitive function, smoking, social participation, and social support. Consistent with previous studies, each of the 15 items was dichotomously rated, and frailty was defined by a summed score of 4 or more points [19,20].

Statistical analysis

Changes associated with the LT intervention were assessed by comparing baseline and follow-up values, and the Wilcoxon signed-rank test was employed alongside McNemar’s test. Participants who maintained a non-LS stage before and after the intervention were excluded from further analyses. The remaining participants were classified into an improvement group (those who showed improvement in the LS stage) and a non-improvement group (those who did not show improvement). Pre-intervention assessment measures and LT implementation frequencies were compared between the two groups. To compare groups, the Mann-Whitney U test and Fisher’s exact test were applied. Statistical analyses were carried out with IBM SPSS Statistics, version 29.0 (IBM Corp., Armonk, NY) with the significance level set at 5%, and effect sizes were calculated.

## Results

Table 1 shows the baseline characteristics.

**Table 1:** Baseline characteristics of the participants Data are presented as the number of participants or medians [25th and 75th percentiles].

Assessment items	Data
Age (year)	76.0 [72.0, 80.5]
Sex	14 females
Height (cm)	149.0 [143.8, 151.8]
Weight (kg)	46.9 [44.9, 59.8]
Body mass index (kg/m^2^)	21.9 [20.4, 27.4]

**Figure 2:**
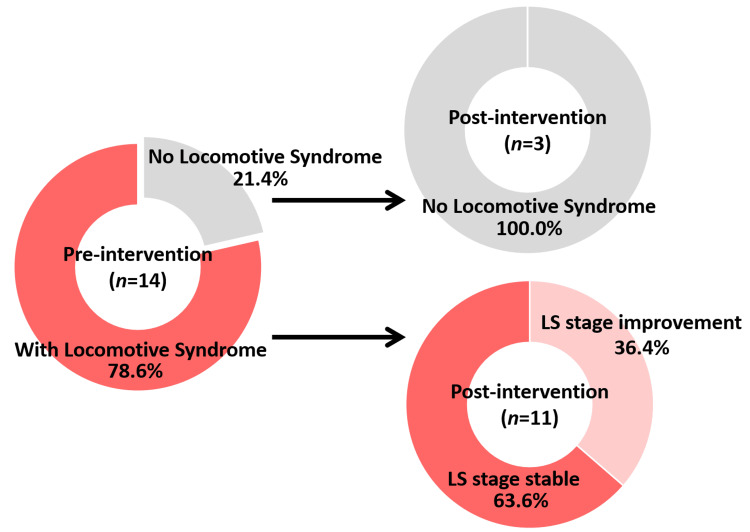
Breakdown of changes in LS stage pre- and post-intervention All three participants who were non-LS pre-intervention maintained their non-LS status post-intervention. Of the four participants who showed improvement in the LS stage after the intervention, one improved from LS stage 3 to 2, one improved from stage 3 to 1, and two improved from stage 2 to 1. The seven participants who maintained their LS stage before and after the intervention were at the LS stage 1. LS, locomotive syndrome

Table 2 shows the changes in the evaluation results before and after the LT intervention. Before the intervention, the LS stages were as follows: three participants were classified as not having LS, seven as LS stage 1, two as LS stage 2, and two as LS stage 3. After the intervention, three participants were classified as not having LS, 10 as LS stage 1, one as LS stage 2, and none as LS stage 3. The distribution of LS stages did not differ significantly between the pre- and post-intervention assessments (p = 0.059). Regarding the transition of LS stages, three participants who were not classified as having LS before the intervention maintained their status. Among the 11 participants who had LS before the intervention, four (36.4%) participants showed improvement in their LS stage, while seven (63.6%) participants maintained their stage. None of the participants experienced worsening of the LS stage (Figure 2). Of the four participants who improved, two showed improvement based on the GLFS-25 score, and two based on the two-step test. The prevalence of frailty was four participants (28.6%) before the intervention and three participants (21.4%) after the intervention. Additionally, the total implementation rate of LT, combining “performed as instructed” and “partially performed” days, was 66.1 ± 10.1 days, corresponding to an adherence rate of 86.9 ± 13.4%.

**Table 2:** Pre- and post-intervention comparison of locomotive syndrome and frailty (n = 14) Data are presented as the number of participants or medians [25th and 75th percentiles]. *p < 0.05 GLFS-25, 25-question Geriatric Locomotive Function Scale; LS, locomotive syndrome; QMCOO, Questionnaire for Medical Check-up of the Old-Old

Variables	Pre-intervention	Post-intervention	Effect size	p-value
Locomotive syndrome				
LS stage (not applicable/1/2/3)	3/7/2/2	3/10/1/0	0.505	0.059
Stand-up test				
Stage (not applicable/1/2/3)	5/9/0/0	5/9/0/0	-	-
Two-step test				
Stage (not applicable/1/2/3)	8/4/1/1	8/5/1/0	0.378	0.157
Two-step test value (cm/height)	1.39 [1.17, 1.41]	1.34 [1.22, 1.46]	0.403	0.132
GLFS-25				
Stage (not applicable/1/2/3)	7/4/2/1	8/5/1/0	0.359	0.180
Total points	6.5 [3.0, 14.3]	4.5 [2.0, 9.3]	0.681	0.011*
Frailty				
QMCOO, ≥4 points	4 (28.6%)	3 (21.4%)	0.267	1.000
QMCOO, total points	1.0 [0.8, 4.0]	2.0 [0.0, 3.3]	0.244	0.361

Among the evaluations of the LS risk test, the GLFS-25 score showed a significant improvement before and after the intervention [pre-intervention: median 6.5 (interquartile range: 3.0-14.3); post-intervention: median 4.5 (2.0-9.3); p = 0.011] (Table 1). Significant improvements were also observed in GLFS-25 item 3 [pre-intervention: median 1.0 (0.0-1.3); post-intervention: median 0.0 (0.0-1.0); p = 0.020] and item 23 [pre-intervention: median 1.0 (0.0-1.0); post-intervention: median 0.0 (0.0-1.0); p = 0.046].

Table 3 presents a comparison of the pre-intervention data and LT implementation frequency between the LS improvement and non-improvement groups. Regarding the pre-intervention LS stage, the improvement group included two participants at the LS stage 2 and two participants at the LS stage 3, whereas the non-improvement group included seven participants at the LS stage 1. The LS stage was significantly more severe in the improvement group than in the non-improvement group (p = 0.003).

**Table 3:** Comparison of baseline data and locomotion training frequency between the LS improvement and non-improvement groups Data are presented as the number of participants or medians [25th and 75th percentiles]. The exercise implementation rate was calculated as the proportion of the total days during which the exercises were either performed as instructed or partially performed. *p < 0.05 LS, locomotive syndrome; QMCOO, Questionnaire for Medical Check-ups of the Old-Old

Variables	LS improvement group (n = 4)	Non-LS improvement group (n = 7)	Effect size	p-value
Age (years)	77.5 [76.0, 81.3]	73.0 [72.0, 80.0]	0.346	0.315
Height (cm)	149.0 [142.0, 150.0]	151.0 [145.0, 158.0]	0.286	0.412
Weight (kg)	46.9 [39.8, 48.1]	58.1 [46.2, 65.6]	0.371	0.230
Body mass index (kg/m^2^)	21.1 [19.5, 21.6]	24.5 [21.7, 28.5]	0.570	0.073
LS				
LS stage (not applicable/1/2/3)	0/0/2/2	0/7/0/0	1.000	0.003*
Frailty				
QMCOO, ≥4 points	2 (50.0%)	2 (28.6%)	0.039	0.576
QMCOO, total points	3.5 [1.5, 5.5]	1.0 [1.0, 4.0]	0.322	0.315
Exercise implementation rate (%)	86.9 [71.1, 97.7]	96.1 [84.2, 100.0]	0.172	0.648

## Discussion

This study examined the changes associated with an LT intervention in a community-based care prevention program conducted in a local municipality. The results showed that the total GLFS-25 score for all participants improved significantly, and among the 11 participants who had LS before the intervention, four (36.4%) participants showed improvement in their LS stage. This study provides valuable evidence supporting the recommendation of LT as an effective and feasible program that is well-suited to real-world community-based care prevention settings, even under constraints of time, personnel, and resources. Importantly, the intervention period in this study was relatively short (76 days) compared with those in previous studies, highlighting the potential of LT to produce meaningful improvements over a limited time frame.

These findings suggest that even short-term exercise interventions can be effective against LS when the program is appropriately structured and continuously implemented. LT is characterized by low exercise volume, simplicity, and ease of memorization, with a clear focus on improving muscle strength and balance. Moreover, because squatting movements induce trunk muscle activity, they may also contribute to improvements in body stabilization [21,22]. Previous studies in Japan have reported exercise adherence rates of 57-75% for programs aimed at promoting exercise continuation, whereas the adherence rates for LT have been reported to exceed 90% [23]. The implementation rate of LT in this study was also high (86.9%), indicating strong adherence to the exercise program. Furthermore, previous studies examining the effects of LT interventions on community-dwelling older adults have reported improvements in GLFS-25 scores [24-26], and the findings of this study support these results.

The characteristics of the participants who showed improvement in their LS stage revealed that they were initially classified as having LS stage 3 or 2. This finding suggests that the effects of exercise interventions vary depending on the severity of LS. Although it is often considered challenging to expect reversible changes in cases of advanced LS (i.e., where mobility decline has progressed), those who showed improvement in this study were among the more severe cases. This supports the possibility that LT can improve musculoskeletal function even in the advanced stages of LS. Such results not only provide evidence from the perspective of LS prevention but also support the efficacy of LT interventions after the progression of musculoskeletal decline. Furthermore, among the 14 participants analyzed, three individuals classified as non-LS before the intervention maintained their status. Of the 11 participants with LS before the intervention, four (36.4%) participants showed improvement in their LS stage. Additionally, none of the participants experienced a worsening of their LS stage after the intervention. These findings indicate that LT may not only prevent the progression of LS but also help prevent its deterioration, demonstrating its effectiveness as a primary and secondary preventive intervention for older adults.

Furthermore, an analysis focusing on individual items of the GLFS-25 revealed significant improvements in items 3 and 23 before and after the intervention. Item 3 is related to pain. It has been reported that low back pain and arthritis in older adults significantly reduce the QOL scores [27]. Additionally, pain has been directly linked to restrictions in social participation [28]. Therefore, the significant improvement in pain-related items following this intervention suggests not only a reduction in physical discomfort but also the potential to contribute to overall QOL improvement through enhanced activity levels and social engagement. Item 23 is related to social participation. A decline in social issues appears first, and social disability has been reported to be associated with important negative outcomes [29]. The observation of some improvement in this social participation indicator even after a relatively short intervention period suggests that LT interventions may have beneficial effects that extend beyond physical function improvement to include social aspects.

Several limitations should be acknowledged in the present study. First, as an exploratory study, this research used a pre-post design without a control group, highlighting the need for future randomized controlled trials to evaluate the effects of LT. Second, the number of participants who completed the follow-up was limited. Participants in the care prevention program were recruited on a first-come, first-served basis through the city’s official website, and due to the nature of the program, there was a limit on the number of participants. Consequently, some individuals were unable to participate in the post-intervention follow-up period. This attrition may have led to potential selection bias, as those who completed the follow-up may have been relatively more motivated or healthier than those who did not. Additionally, the small sample size limited the statistical power, making the effect size estimates less stable and the generalizability of the findings restricted. In addition, because this study was conducted as part of a care prevention program, detailed information on participants’ medical histories and comorbidities could not be collected. Third, all the analyzed participants were women. Given the extremely small number of male participants in this preventive care program, the analysis was limited to female participants. Low participation of men in preventive care programs has similarly been noted in other municipalities [30]. In the future, establishing a system that allows continuous tracking of a larger number of participants, while accounting for clinically meaningful changes, is expected to build evidence with higher validity.

## Conclusions

This study examined the changes associated with LT as an exercise intervention for LS in older women living in the community and involved in a care prevention program implemented by a local municipality. Among the 11 participants who had LS before the intervention, four (36.4%) showed improvement in their LS stage, and significant improvements were observed in the GLFS-25 scores, including items related to pain and social participation. These findings suggest that LT may be an effective exercise intervention for improving musculoskeletal function in older adults, even when implemented within the limited time and resources of care prevention programs.
